# Special Issue “Advances in Rabies Research”

**DOI:** 10.3390/v15071557

**Published:** 2023-07-16

**Authors:** Susan A. Nadin-Davis

**Affiliations:** Ottawa Laboratory Fallowfield, Canadian Food Inspection Agency, Ottawa, ON K2H 8P9, Canada; nadindavis@gmail.com

Rabies kills approximately 60,000 humans each year, with deaths mostly occurring in developing countries, where rabies lyssavirus (RABV) variants are maintained in dog populations. However, RABV is merely one of several viral species comprising the *Lyssavirus* genus, most of which are maintained in bat species, and all of which are potential etiological agents of this disease. Surveillance and molecular epidemiological studies have expanded our knowledge of the genetic diversity and geographical range of lyssaviruses and allowed us to identify their reservoir hosts. Continuing development of diagnostic tools, improved vaccination strategies and better understanding of the virus–host relationships that influence infection outcome will all contribute to future success of rabies control strategies. This Special Issue presents 12 peer-reviewed reports that describe research related to these areas of study.

Timely diagnosis and disease surveillance is pivotal to effective rabies control. Strengthening developing countries’ rabies management capabilities is a priority of the Zero By 30 initiative, which seeks to eliminate cases of dog-mediated human rabies by 2030. Accordingly, the World Organization for Animal Health has supported several related laboratory twinning initiatives. Such a collaboration between the Friedrich-Loeffler-Institut, Germany, and the Central Veterinary Laboratory, Windhoek, Namibia, describes the application of multiple testing regimens, including the direct fluorescent antibody (DFA) test, multiple RT-PCRs and the feasibility of the field use of lateral flow devices [1]. While the DFA test remains the gold standard method of rabies diagnosis, real-time quantitative RT-PCR (RT-qPCR) tests, which are potentially more sensitive than traditional tests, have gained increased acceptance. Indeed, the value of the LN34 pan-lyssavirus RT-qPCR assay for confirmatory RABV testing of samples initially considered to be DFA negative is reported, with a cautionary note regarding the importance of testing multiple brain regions to ensure accurate diagnosis [2]. Although RT-qPCRs for RABV detection are well developed, the continuing expansion of the known genetic diversity of the *Lyssavirus* genus makes the design of pan-lyssavirus protocols more challenging. One study identifies deficiencies in the scope of four widely used pan-lyssavirus RT-PCRs and describes a modified LN34 RT-qPCR assay that improves lyssavirus range [3]. Participation in proficiency testing (PT) helps rabies-testing laboratories to maintain high diagnostic accuracy. Generation of PT panels for use in the RABV DFA test requires significant quantities of well-standardized rabies-positive tissues, which are usually generated via viral propagation in large numbers of intracerebrally inoculated mice. To address resulting animal welfare concerns, an opinion piece describes alternative methods used to compile PT panels for use in both DFA and RT-PCR testing, which apply the 3Rs (Replace, Reduce, Refine) [4].

DNA sequencing advancements have transformed our capability to characterize lyssaviruses using material extracted from fresh or frozen tissues; however, their application to formalin-fixed paraffin-embedded (FFPE) samples remain challenging. The application of an Ampliseq methodology to enable amplification and sequencing of large portions of the genomes of a wide variety of RABV variants is reported [5]. The application of this protocol to enable detailed phylogenetic analysis of RABV in FFPE samples provides a molecular epidemiological tool for analyzing the virus in historical fixed samples.

Vaccines play critical roles in rabies prevention, both as inactivated vaccines to protect humans and companion animals and as attenuated live vaccines used for oral vaccination in wildlife and feral dogs. The complete genome characterization and phylogenetic analysis of RABV strains employed for production of inactivated vaccines in Japan, together with several progenitor strains, generally confirm their recorded relationships and explore the accompanying viral coding changes [6]. Highly parallel sequencing of the viral genome of a replication-competent rabies vaccine—SPBN GASGAS—during serial passage in cell culture and suckling mice is described; deep sequence coverage enables assessment of the stability of the vaccine’s consensus sequence, as well as the nature and extent of low-level variants [7]. As the high level of attenuation in this strain precludes its serial passaging in target species to demonstrate lack of virulence, as dictated by European Union licensing requirements, such genetic stability evaluations could, if subjected to appropriate regulatory guidelines, contribute to verification of the product’s safety.

Monitoring rabies vaccination campaigns in dog populations and evaluating the factors that impact their success is an important component of disease control, and the application of this approach is described for the Changsha region of China over a six-year period [8]. Long-term protection from rabies is provided by IgG rabies virus neutralizing antibodies that target viral glycoprotein. These reagents can be induced in humans at high risk of rabies exposure via pre-exposure prophylaxis (PrEP) involving a three-shot rabies vaccination regimen. A novel study of a small human cohort that received PrEP explores the kinetics of antibody isotype switching from anti-rabies IgM to IgG, knowledge which may guide future refinement of vaccination regimens [9].

Modelling studies that combine disease surveillance data and advanced molecular epidemiological analysis have yielded significant insights regarding the factors that impact RABV maintenance in host reservoirs and persistence following transmission to new host species. Expanding upon previous studies of RABV circulation in North American bats, the most important factor that influences the virus’ spread between *Myotis* bat species is range overlap, in contrast to the important role of host genetic diversity in constraining transmission between different bat genera [10]. Another study explores the use of a spatially explicit agent-based computer model, which incorporates aspects of host and pathogen biology and different control scenarios to simulate spatio-temporal rabies dynamics across Southern Ontario following the introduction of raccoon rabies [11]. Although such models can predict disease outcomes under many different scenarios, thereby informing rabies wildlife control programs, accounting for all factors that impact disease transmission in this field is still challenging.

A study that aims to better understand the neurotropic nature of RABV pathogenesis concludes that complex interplay between several cell types, including neurons, microglia and astrocytes, that involve immune responses and cytokine circulation, plays a critical role in determining the outcome of viral infection [12].

The wide scope of these reports highlights the importance of applying a broad range of research tools to combat rabies in a One Health context. Future success will require accurate and rapid diagnosis at a global level, better understanding of viral transmission patterns, availability of efficacious and safe vaccines for both humans and reservoir hosts and improved knowledge of the mechanisms of viral pathogenesis to enable the development of effective patient treatment strategies.
